# Daily locomotor activity declines with tumor growth and disease progression in glioblastoma

**DOI:** 10.1172/jci.insight.194582

**Published:** 2025-11-25

**Authors:** Maria F. Gonzalez-Aponte, Sofia V. Salvatore, Anna R. Damato, Ruth G.N. Katumba, Grayson R. Talcott, Omar H. Butt, Jian L. Campian, Jingqin Luo, Joshua B. Rubin, Olivia J. Walch, Erik D. Herzog

**Affiliations:** 1 Department of Biology, Division of Biology and Biomedical Sciences, Washington University in St. Louis, St. Louis, Missouri, USA.; 2 Division of Oncology, Department of Medicine, Washington University School of Medicine, St Louis, Missouri, USA.; 3 Department of Oncology, Mayo Clinic, Rochester, Minnesota, USA.; 4 Division of Public Health Sciences, Department of Surgery,; 5 Siteman Cancer Center Biostatistics and Qualitative Research Shared Resource,; 6 Department of Pediatrics, St. Louis Children’s Hospital, and; 7 Department of Neuroscience, Washington University School of Medicine, St. Louis, Missouri, USA.; 8 Arcascope Inc., Arlington, Virginia, USA.; 9 Department of Neurology, University of Michigan, Ann Arbor, Michigan, USA.

**Keywords:** Clinical Research, Neuroscience, Oncology, Behavior, Brain cancer, Clinical practice

## Abstract

Glioblastoma (GBM) is an aggressive brain tumor that often progresses despite resection and treatment. Timely and continuous assessment of GBM progression is critical to expedite secondary surgery or enrollment in clinical trials. However, current progression detection requires costly and specialized MRI examinations, which, in the absence of new symptoms or signs, are usually scheduled every 2–3 months. Here, we hypothesized that changes in daily activity are associated with GBM growth and disease progression. We found that wheel-running activity in GBM-bearing mice declined as tumors grew and preceded weight loss and circadian breakdown by over a week. Temozolomide treatment in the morning, but not evening, significantly reduced tumor size and restored daily locomotion in mice. In a pilot study of 6 patients with GBM wearing an actigraphy watch, wrist movement provided a feasible and continuous longitudinal indicator of daily activity with 1-minute resolution. After tumor resection and radiation, daily activity declined in 2 patients 19 and 55 days before detection of progression by MRI but did not change for the 4 patients with stable disease. These results suggest that daily activity tracking using wearable devices may serve as a real-time indicator and potential monitoring tool for GBM progression and treatment efficacy.

## Abstract 2

Monitoring daily activity correlates with glioblastoma growth and enables earlier progression detection, offering a real-time, non-invasive monitoring tool for glioblastoma progression to advise timely interventions.

## Graphical abstract

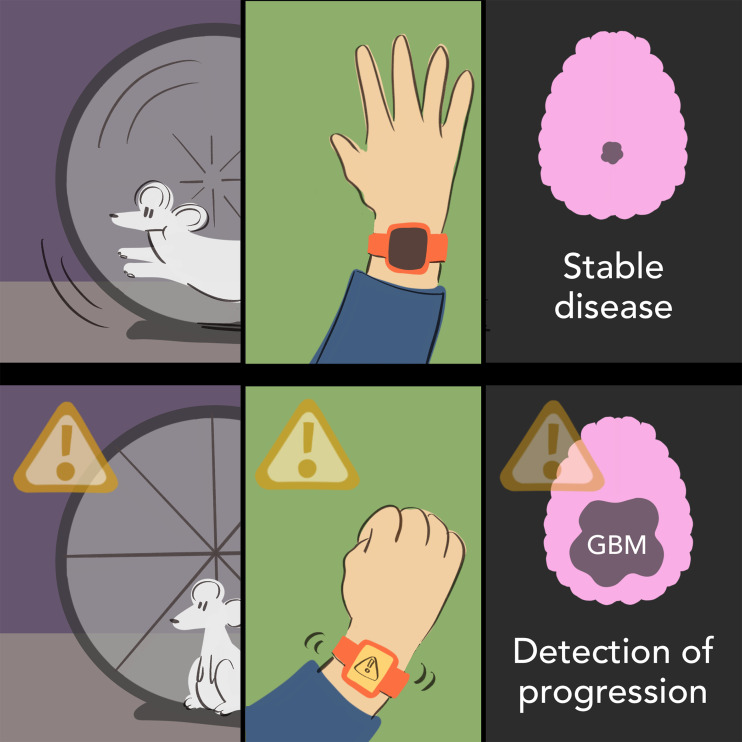

## Introduction

Glioblastoma (GBM) is among the most aggressive primary malignant brain tumors in adults, accounting for 50% of all brain tumor deaths (1, 2). Detection of primary, or recurrent, GBM often occurs after the development of chronic headaches, progressive neurological deficits, seizures, sleep disruption, and others factors (3–11). Diagnosis and detection of progression currently involve the use of brain imaging scans such as CT or MRI, which are typically scheduled every 2–3 months (4). These tumors typically present as an irregularly shaped mass with a dense ring of enhancement and a necrotic center that appears hypointense (12). Imaging scans may also reveal surrounding vasogenic edema, hemorrhage, and distortion of the ventricles (13, 14). After diagnosis, the standard of care for patients with GBM consists of maximal safe surgical resection, followed by concurrent radiation and chemotherapy with temozolomide (TMZ), and lastly, adjuvant chemotherapy with TMZ (15). Phase III clinical trials have demonstrated that tumor-treating fields, together with resection, irradiation, and TMZ, prolong survival in newly diagnosed GBM cases. Thus, tumor-treating fields have increasingly become a component of up-front care (16). Despite these aggressive treatment approaches, median survival for newly diagnosed GBM is around 9–16 months, depending on factors like age, sex, and treatment. Approximately 70% of patients will have recurrence within 1 year of surgical resection (1, 17, 18).

Depending on location, GBM can extensively grow and invade before detection. In murine models and patients, GBM progression often involves a prolonged asymptomatic period even for tumors of a large size, followed by a rapid and severe decline in clinical condition (19–21). In preclinical murine models, body weight loss has been the standard measurement of disease progression (22, 23). However, although a rapid decline in body weight of 20% has served as a predictor of clinical deterioration in some studies (22, 24), others have suggested it is unreliable (19, 23). Other qualitative metrics for disease progression in rodents, like nesting and feeding behaviors, have been considered, but interobserver variability has limited their utility (20, 24).

In patients with GBM, brain imaging techniques, typically scheduled every 2–3 months after tumor resection, are the primary approach to monitoring GBM progression. However, distinguishing between true progression versus pseudo-progression due to treatment changes remains an ongoing challenge that delays detection of progression and timely interventions (13, 14). Moreover, monitoring disease progression every 2–3 months lacks the expediency needed with an aggressive brain tumor that has a median survival of 3 to 9 months after recurrence (1, 17, 18). Timely detection of disease progression with alternative methods, such as wearables, mobile apps, and real-time tracking devices, may address limitations presented by current standard-of-care interval scans. Real-time and continuous detection of disease progression could lead to interventions that improve outcomes, including secondary resections (when possible) and enrollment in clinical trials, which are often only available to patients with recurrent GBM. Further, timely detection of GBM progression could point to interventions that improve the quality of life and palliative care of patients before death, in cases where surgical resection or participation in clinical trials is not an option. Alternative biomarkers and real-time monitoring tools can complement brain imaging scans. Wearable devices may have the potential to track disease progression and recurrence in GBM. Actigraphy watches, smartwatches, mobile apps, and fitness trackers are noninvasive, cost-effective, and patient friendly, and they provide continuous and accurate recording of physical activity, sleep patterns, and physiological parameters (25, 26). Recent studies have found that tracking sleep-wake cycles with actigraphy devices can serve as a predictor of progression and survival in patients with breast, lung, or prostate cancer, but this remains to be evaluated in GBM (27–31). In all the aforementioned cancer types, better sleep efficiency and decreased disruption of sleep-wake cycles measured by wrist activity was found to predict a significant reduction in overall mortality and cancer risk. The integration of wearable technology into clinical practice could therefore enable longitudinal, real-time monitoring of patients, which may facilitate the detection of GBM progression.

Here, we test the hypothesis that changes in daily activity are associated with GBM growth and may indicate disease progression earlier than standard measurements. Altogether, we found that activity declines with GBM growth in tumor-bearing mice and before clinical detection of progression in patients. We propose that actigraphy can be further evaluated as a real-time monitoring tool for timely detection of GBM progression.

## Results

### *Daily locomotor activity declines with GBM growth and disease progression*.

**Figure 1:**
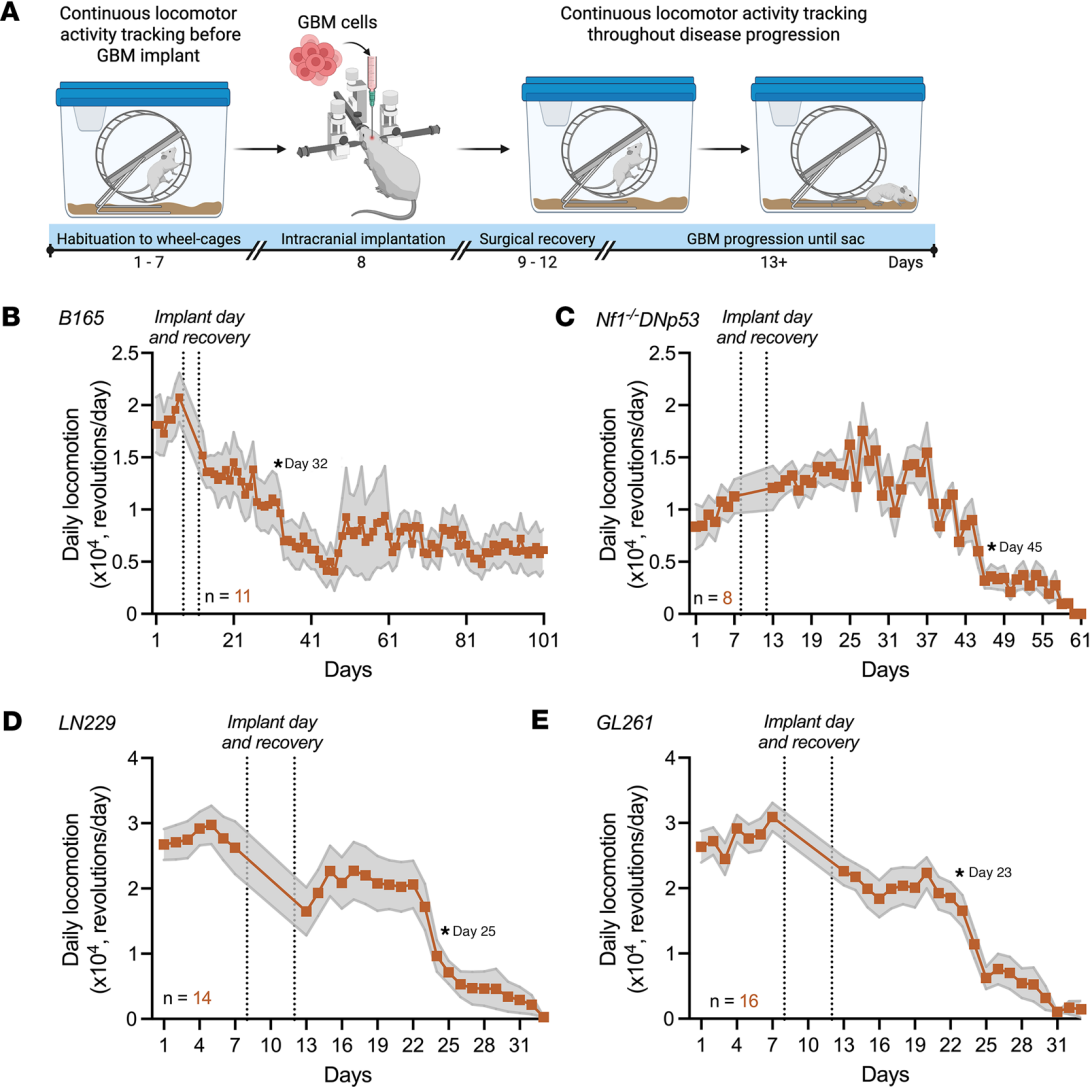
Daily locomotor activity declines with tumor progression in GBM-bearing mice. (**A**) Schematic of locomotor activity recording before and through GBM progression. We recorded wheel-running activity continuously from mice for 7 days before and continuously through disease progression after GBM implant into the basal ganglia. (**B**) Daily locomotor activity of mice bearing patient-derived B165 tumors significantly declined 24 days after implant (mean ± SEM, *n* reported in figure, asterisk indicates day of 10% locomotion decline, time series change point detection analysis, *P < 0.05). Dotted lines on days 9–12 indicate dates of surgical recovery when locomotor activity was not recorded. (**C**) Daily locomotor activity of mice bearing syngeneic murine Nf1^–/–^DNp53 tumors significantly declined 37 days after implant (mean ± SEM, *n* reported in figure, asterisk indicates day of 10% locomotion decline, time series change point detection analysis, *P < 0.05). Dotted lines on days 9–12 indicate dates of surgical recovery when locomotor activity was not recorded. (**D**) Daily locomotor activity of mice bearing human LN229 tumors significantly declined 17 days after implant (mean ± SEM, *n* reported in figure, asterisk indicates day of 10% locomotion decline, time series change point detection analysis, *P < 0.05). Dotted lines on days 9–12 indicate dates of surgical recovery when locomotor activity was not recorded. (**E**) Daily locomotor activity of mice bearing murine GL261 tumors significantly declined 15 days after implant (mean ± SEM, *n* reported in figure, asterisk indicates day of 10% locomotion decline, time series change point detection analysis, *P < 0.05). Dotted lines on days 9–12 indicate dates of surgical recovery when locomotor activity was not recorded.

To identify a biomarker that can be measured more often and easily than standard-of-care MRI in GBM, we tested whether GBM growth impairs daily locomotion. First, we evaluated the impact of orthotopic GBM xenografts on running-wheel activity in mice. To evaluate locomotion in diverse preclinical models of GBM, we implanted primary patient-derived (B165), human (LN229), or murine (Nf1^^^–/–^^^DNp53 and Glioma 261 [GL261]) cells. These well-characterized models of GBM have been found to have reliable circadian rhythms in clock gene expression (32). We next tracked daily wheel-running activity throughout disease progression (Figure 1A). Daily locomotor activity significantly declined as the disease progressed across all GBM models (Figure 1, B–E, time series change point detection analysis, *P < 0.05). On average, daily locomotion declined by at least 10% on days 23, 25, 32, and 45 in mice bearing GL261, LN229, B165, and Nf1^^^–/–^^^DNp53 tumors, respectively (Figure 1, B–E).

**Figure 2:**
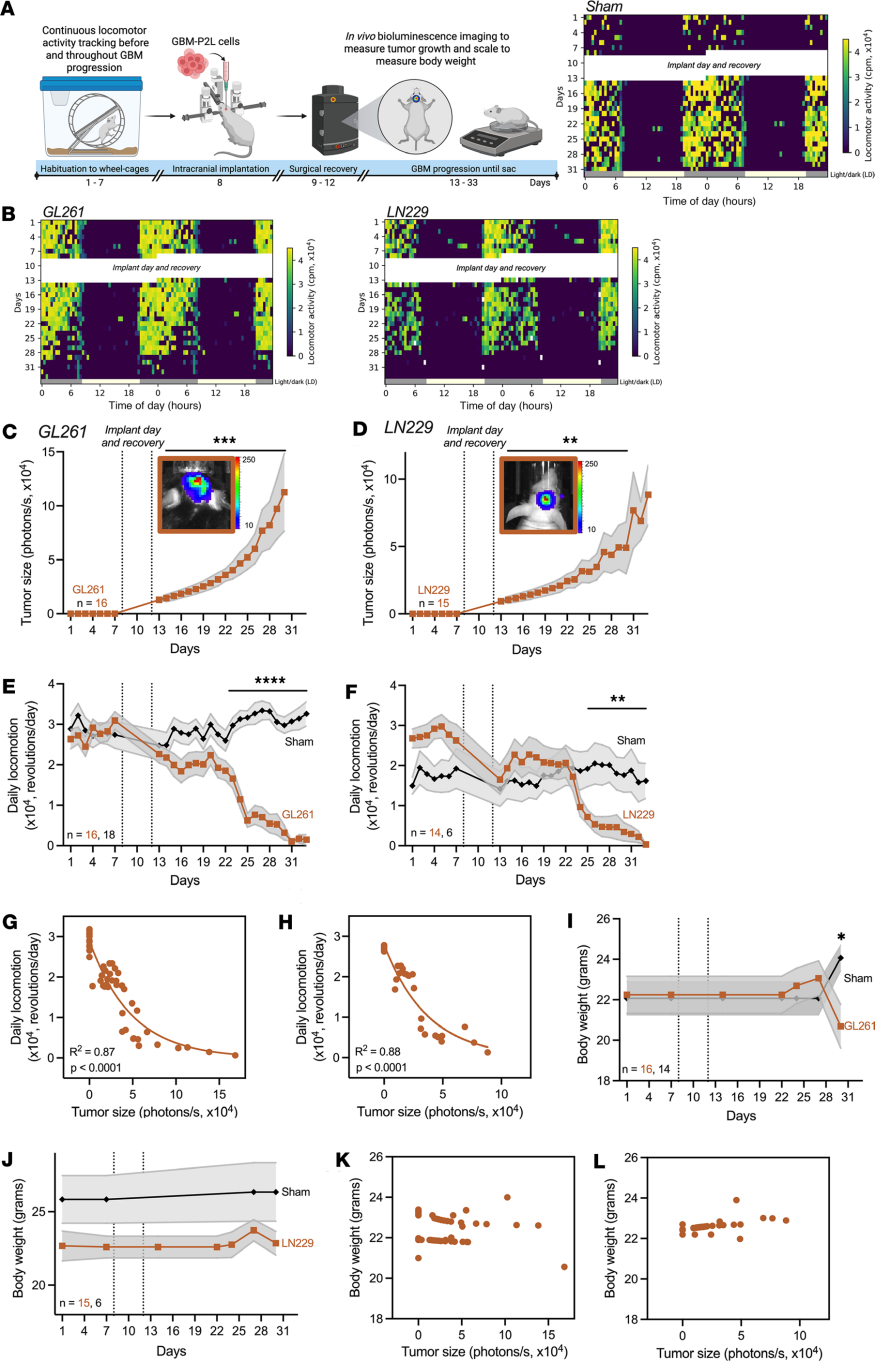
Daily locomotion declined with intracranial tumor growth. (**A**) We recorded running-wheel activity for before and after implantation of GL261 or LN229 cells into basal ganglia. Representative locomotion in a sham mouse was stable throughout recording. Yellow and gray bars represent day and night (lights on 7 a.m. to 7 p.m.). (**B**) Actograms before and after GL261 (left) or LN229 (right) tumor implantation. Note daily locomotion declined as disease progressed. (**C** and **D**) Tumor bioluminescence increased 9-fold from 5 to 22 days after implant in mice bearing GL261 or LN229 tumors (mean± SEM, 1-way repeated-measures ANOVA with Tukey’s multiple-comparison test, **P < 0.01, ***P < 0.001). Dotted lines indicate surgical recovery (days 9–12). Inset images show tumor bioluminescence 22 days after implant. (**E** and **F**) Daily locomotion of GBM-bearing mice declined 15 or 17 days after implant compared with sham (mean ± SEM, 2-way repeated-measures ANOVA with Tukey’s multiple-comparison test, **P < 0.01 from days 25 to 33, ****P < 0.0001 from days 23 to 33). (**G** and **H**) Average daily locomotion declined as GBM tumors grew (GL261: Spearman’s correlation *r* = –0.92, 95% CI: –0.95 to –0.86, *P* < 0.0001, line shows nonlinear regression fit; LN229: *r* = –0.92, 95% CI: –0.97 to –0.84, *P* < 0.0001). (**I** and **J**) Body weight declined 22 days after implant in GL261-, but not LN229-, bearing mice compared with controls (mean ± SEM, 2-way ANOVA with Tukey’s multiple-comparison test, *P < 0.05). (**K** and **L**) Weight did not correlate with GL261 (*r* = –0.25, 95% CI: –0.50 to 0.03, *P* = 0.07) or LN229 tumor size (*r* = –0.15, 95% CI: –0.36 to 0.12, *P* = 0.06). Each dot represents average of all mice per recording day, excluding days of surgery and recovery.

Taking advantage of the ability to track rapid disease progression with immortalized, infiltrative GBM models, we tracked daily tumor size by in vivo bioluminescence imaging, body weight, and locomotion after implanting GL261 or LN229 GBM cells expressing a *Per2* luciferase reporter (GBM-P2L), which have previously been found to have reliable circadian rhythms and integrate into the brain’s circadian circuit to grow (Figure 2, A and B) (32). We found that GBM tumor size increased by an average of 9-fold from 5 to 22 days after implant in GL261- and LN229-bearing mice (Figure 2, C and D). Concurrently, daily activity of GBM-bearing mice significantly declined by 22% ± 4% (mean ± SEM) starting on days 15 (GL261) and 17 (LN229) after implant, compared with sham-operated controls (Figure 2, E and F), when tumors reached a size of 3.3 × 10^^^4^^^ ± 0.6 × 10^^^4^^^ photons/s (mean ± SEM, Figure 2, C and D). Tumor size highly inversely correlated with daily locomotor activity counts (GL261: Spearman’s rank *r* = –0.92, 95% CI: –0.95 to –0.86, *P* < 0.0001; LN229: Spearman’s rank *r* = –0.92, 95% CI: –0.97 to –0.84, *P* < 0.0001). Nonlinear regression analysis showed a significant negative correlation between tumor size and locomotor activity, suggesting that as GBM tumors grow, locomotor activity declines (Figure 2, G and H, and Supplemental Figure 1, A and B; supplemental material available online with this article; https://doi.org/10.1172/jci.insight.194582DS1). In contrast, while GBM-bearing mice lost an average of 10% of their body weight just before death (Figure 2, I and J), we found tumor size did not correlate with body weight (Pearson and Spearman’s rank correlation coefficient tests, *P* > 0.05, Figure 2, K and L). Altogether, these results suggest that daily locomotion declines earlier than other standard indicators of disease progression and significantly correlates with tumor growth in preclinical GBM models.

Consistent with prior studies (33–35), GL261 GBM tumors implanted into male hosts grew significantly larger than in females toward the end of the disease (Supplemental Figure 2, A and B). However, regardless of host sex, locomotor activity significantly declined (by 30% ± 6% in males, 14% ± 3% in females; mean ± SEM) around day 15 after implant compared with controls when GL261 tumors reached a size of 4 × 10^^^4^^^ ± 1.2 × 10^^^4^^^ photons/s in males and 3 × 10^^^4^^^ ± 0.5 × 10^^^4^^^ photons/s in females (mean ± SEM, *P* > 0.05, 2-tailed Student’s *t* test, Supplemental Figure 2, C and D). In both males and females, tumor size inversely correlated with locomotor activity counts (Supplemental Figure 2, C and D) but not with body weight (Supplemental Figure 2, E and F). These results suggest that although sex differences exist in tumor size toward the end of disease and in the percentage of locomotor activity lost, detecting a decline in daily locomotion occurs at a similar stage of GBM progression in both sexes.

To evaluate whether other characteristics of locomotion change with GBM progression, we measured wake onset time, rest onset time, circadian amplitude, and sleep regularity index (SRI) in mice with and without GBM xenografts. We found that compared with sham controls, GBM-bearing mice woke up earlier (Supplemental Figure 3, A and B) and initiated rest later (Supplemental Figure 3, C and D) toward the end of the disease and regardless of host sex. However, these parameters changed late in the disease, and no significant correlations were found between tumor size and wake onset or rest onset times (Supplemental Figure 3, A–D). Similarly, circadian amplitude and SRI declined late in the disease, weakly correlating with GBM growth in both males and females, suggesting impairment of the circadian system just before death (Supplemental Figure 3, E–H).

### *Morning chemotherapy with TMZ reduces GBM growth and restores locomotor activity loss*.

**Figure 3:**
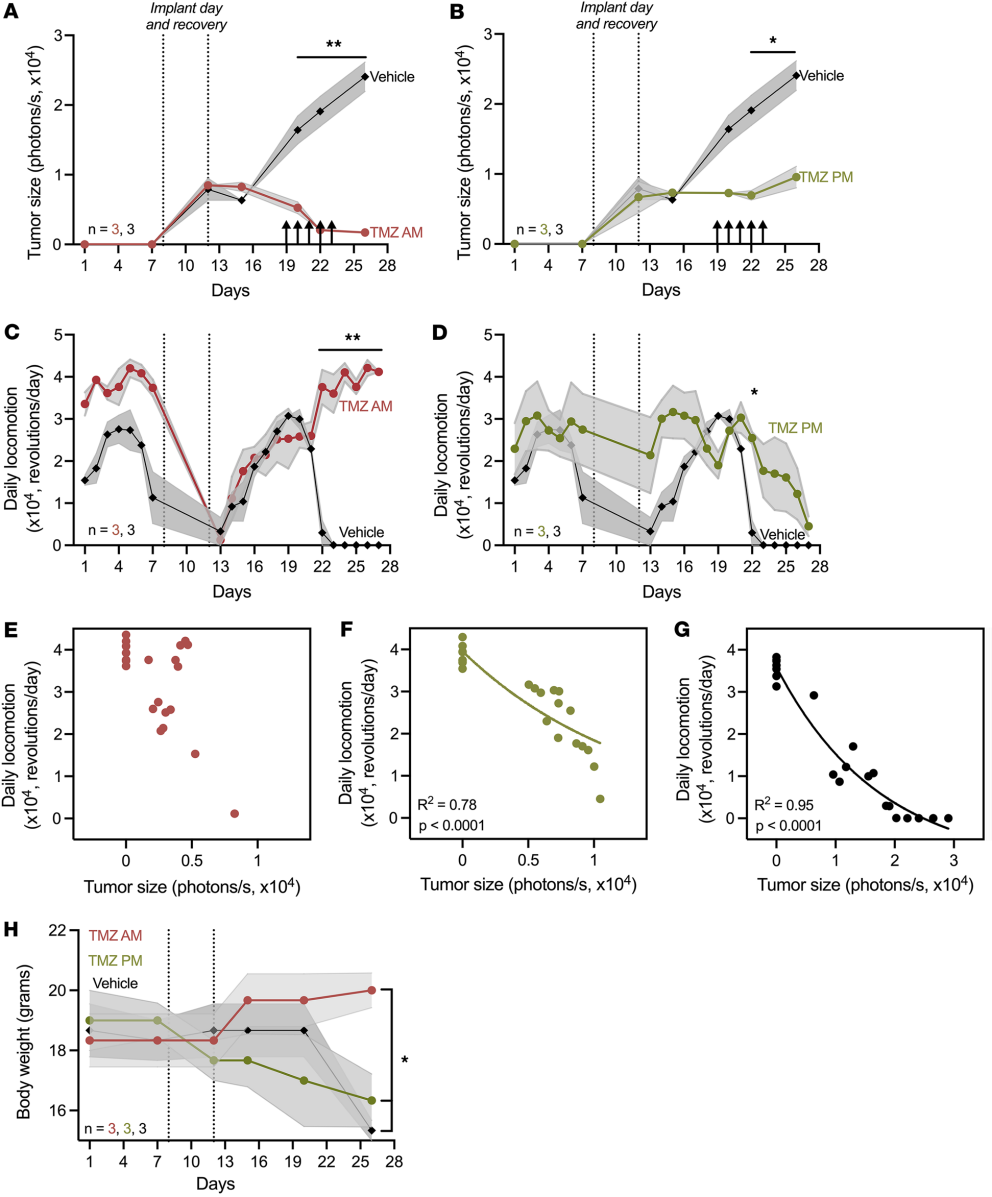
Delivering TMZ in the morning reduced GBM tumor size and restored baseline daily locomotor activity. (**A** and **B**) GL261 xenografts decreased in size when treated with morning (**A**) or evening TMZ (**B**) com¬pared with vehicle (AM: **P < 0.01 from days 20 to 26; PM: *P < 0.05 from days 22 to 26; mean ± SEM, 2-way ANOVA with Tukey’s multiple-comparison test). Arrows indicate days of TMZ or vehicle treatment. (**C** and **D**) Mice receiving morning (**C**) or evening (**D**) TMZ recovered locomotion compared to vehicle (*P < 0.05; **P < 0.01; Mean ± SEM, 2-way ANOVA with Tukey’s multiple-comparison test). (**E** and **F**) Daily locomotion did not correlate with tumor size in mice receiving morning TMZ (*r* = –0.34, 95% CI: –0.69 to 0.11, *P* = 0.12) but negatively correlated with tumor size in mice receiving evening TMZ (*r* = –0.94, 95% CI: –0.98 to –0.87, *P* < 0.0001). Each dot represents daily average of each mouse, excluding days of surgery and recovery. (**G**) Daily locomotion negatively correlated with tumor size in vehicle-treated mice (*r* = –0.95, 95% CI: –0.98 to –0.87, *P* < 0.0001). (**H**) Body weight declined 18 days after implant in mice treated with vehicle or evening TMZ and increased in mice treated with morning TMZ (mean ± SEM, 2-way ANOVA with Tukey’s multiple-comparison test, *P < 0.05 TMZ am vs. TMZ pm, and TMZ am vs. vehicle).

To evaluate whether actigraphy-based scoring of GBM progression is robust to chemotherapy-induced changes in behavior and tumor growth, we recorded locomotor activity, tumor bioluminescence, and body weight in GBM-bearing mice treated with vehicle or TMZ in the morning or evening. Consistent with prior studies, tumor size declined more in mice treated with TMZ in the morning, compared with evening and vehicle treatment (Figure 3, A and B) (36). We found that TMZ in the morning resulted in daily locomotion returning to baseline, whereas TMZ in the evening or vehicle treatment failed to rescue locomotor activity (day 14 after implant, Figure 3, C and D). Total daily locomotion inversely correlated with tumor size in mice receiving TMZ in the evening or vehicle but was uncorrelated with tumor size in mice treated in the morning (Figure 3, E–G). Non-linear regression analysis showed a significant negative correlation between tumor size and locomotor activity in mice treated with TMZ in the evening or with vehicle, suggesting that as GBM tumors continue to grow, daily locomotor activity declines (Figure 3, F and G). In parallel, we found that body weight increased for mice treated with TMZ in the morning but decreased in mice treated with TMZ in the evening or with vehicle on day 18 after implant and toward the end of the disease (Figure 3H). Together, these results demonstrate that morning administration of TMZ induced maximum GBM tumor death and reversed locomotor decline, suggesting that daily activity loss can be restored with chemotherapy and that TMZ efficacy can be monitored with activity tracking.

### *Wrist activity detects disease progression in patients with GBM*.

**Figure 4.**
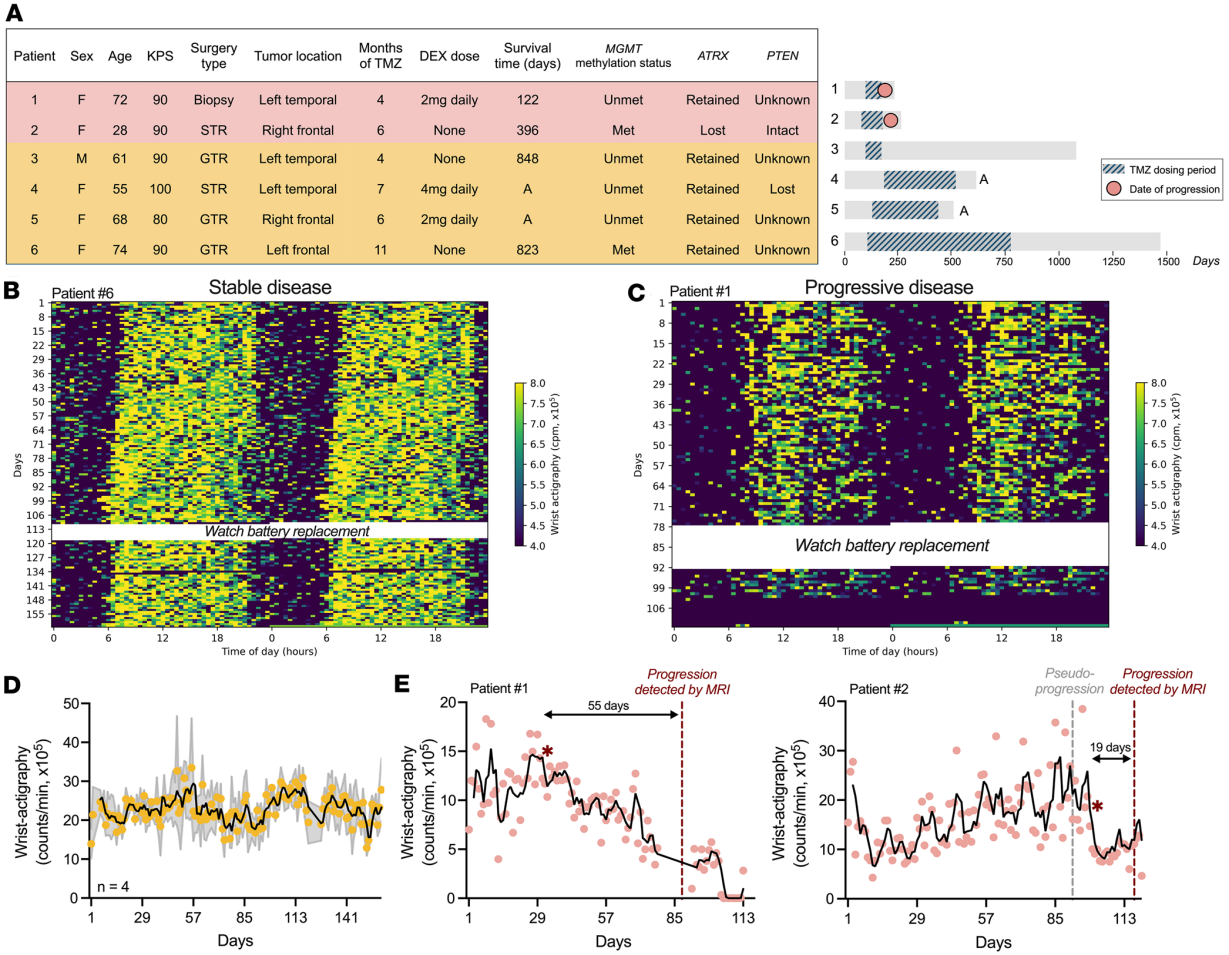
Daily activity declined before clinical detection of GBM progression in patients. (**A**) (Left) Patients with GBM continuously wearing wrist actigraphy watches characterized by sex, age, Karnofsky Performance Status (KPS) score, surgery type (STR, subtotal resection; GTR, gross total resection or biopsy), tumor location, months of TMZ treatment, dexamethasone (DEX) steroid dose, survival time, MGMT promoter methylation status (Unmet, unmethylated; Met, methylated), and ATRX and PTEN expression status. (Right) After surgery and radiation on day 0, wrist actigraphy (gray bars) was recorded for patients receiving TMZ for 5 days every 21 days while being monitored for tumor progression (circles). “A” indicates patient was alive when actigraphy data collection was finalized for all trial participants. (**B**) Representative actogram of a patient (#6) with stable disease (i.e., did not progress during study) and reliable daily activity pattern and level. Blue-yellow colors depict wrist activity counts and white indicates missing data (e.g., Actiwatch required battery replacement). (**C**) Representative actogram of patient (#1) with progressive dis¬ease (i.e., tumor size increased on scans during study) wearing actigraphy watch after completing surgery and during maintenance TMZ treatment. Note decline in daily activity. (**D** and **E**) Patients without disease progression showed stable daily locomotion (mean ± SEM, black line indicates 3-day running average of daily activity data, time series change point detection analysis; *P* > 0.05), while the 2 who progressed showed a sustained 10% decrease in wrist movements on days 33 (#1, 55 days before clinical detection of progression) and 98 (#2, 19 days before clinical detection of progression, asterisk indicates time-series change-point detection analysis, *P < 0.05). Arrows indicate times of activity decline and MRI detection of progression. Vertical lines show dates of MRI-based disease progression (red) or pseudo-progression (gray). See also Supplemental Figures 4 and 5.[Sec sd]

To assess the potential clinical application of actigraphy to monitor GBM progression, we analyzed wrist-based activity and clinical data from patients with primary brain tumor who continuously wore a motion watch as part of a prospective clinical trial that randomly assigned patients to take TMZ in the morning or evening (Figure 4A) (37, 38). In this small-cohort pilot study, wrist movements were recorded every minute after surgery and radiation and through disease progression. Patients underwent treatment cycles of daily TMZ delivered for 5 days every 21 days and were monitored for tumor progression or pseudo-progression by MRI approximately every 8 weeks. Upon detection of progression, patients returned the watch and left the study to pursue additional treatment options. Of the 10 patients originally enrolled in the study, 4 were excluded from analysis given a non-GBM diagnosis (i.e., 2 patients diagnosed with *IDH* mutant gliomas, 1 with astrocytoma, and 1 with anaplastic oligodendroglioma). Of the 6 remaining primary patients with GBM, 4 did not progress while on the study (i.e., tumor growth was not detected on imaging scans) and 2 progressed (i.e., tumor growth was detected on imaging scans) (Figure 4A).

To establish the feasibility of incorporating actigraphy watches into clinical care, we analyzed patient compliance and found that it was excellent, with no complaints or device-related adverse events. Patients who received a watch during the study continuously wore it for an average of 22 months (range 8–49 months), corresponding to their length of time in the study. Wrist movements were recorded continuously during the patients’ enrollment in the trial, except for days when watches lost battery and required a charged replacement (approximately 11 days total). Next, we found that patients without disease progression had higher and more stable wrist activity counts compared with those who progressed during the study (Figure 4, B–D, and Supplemental Figure 4, A and B). In contrast, wrist movements declined in the 2 patients with GBM who progressed during the study and had shorter survival times (Figure 4, A and E). Using a threshold of 10% decline, daily wrist activity counts decreased in 2 patients with GBM 19 and 55 days prior to clinical detection of progression with MRI, respectively. These data demonstrate that daily activity declines before detection of progression by routine MRI, highlighting its potential as a feasible and continuous indicator of GBM progression.

To further evaluate whether daily patterns of activity change with GBM progression, we analyzed wake and rest onset times, circadian amplitude, and sleep regularity in all patients. We found that wake onset time did not significantly change in patients with stable disease, but delayed (i.e., patients woke up later in the day) in patients after clinical progression was detected (Supplemental Figure 5, A and B). Moreover, daily rest onset time did not significantly change in patients with stable disease, but advanced (i.e., patients went to bed earlier) in patients who progressed around the time when clinical progression was detected (Supplemental Figure 5, C and D). Analysis of circadian amplitude showed no significant changes over the course of disease in patients with stable or progressive GBM (Supplemental Figure 5, E and F). Lastly, we found a significant 10% decline in SRI around 15 days after detection of clinical progression in 1 patient with GBM (Supplemental Figure 5, G and H). Altogether, these results suggest that the robustness of circadian rhythms in activity may change late in GBM progression in some patients. We conclude that total daily activity, measured continuously with actigraphy watches, declines as GBM progresses.

## Discussion

Our findings demonstrate that daily locomotor activity declines with GBM progression, and it is significantly associated with tumor growth in both male and female mice. Preclinical models historically have relied on body weight loss as an indicator of GBM progression (19, 20, 22, 23). However, in our study, weight loss did not correlate with tumor growth until later in the disease course. Furthermore, we found that daily locomotion, not body weight loss, was rescued when TMZ treatment reduced GBM growth. We propose that daily locomotor activity loss provides a real-time, early indicator of GBM progression and regression in mice.

Given this promising role of daily locomotor activity in preclinical modeling, we next completed a proof-of-concept pilot study examining the feasibility of recording wrist actigraphy in patients undergoing standard-of-care treatment for primary GBM. The current clinical standard to determine progression derives from a combination of clinical and radiographic changes, delineated in standardized criteria such as RANO and RECIST. Despite advances in neuroimaging that better distinguish between healthy brain tissue and residual tumor, distinction between true- and pseudo-progression remains an ongoing challenge (39–42). Further, asymptomatic tumor progression can be hard to detect when scans are obtained every 2–3 months and when clinical qualitative exams are mainly assessed only during in-person monthly assessments. Our study shows that wrist movements can be easily and reliably obtained using a small, noninvasive device compatible with patient quality of life and independence (37). Patient compliance was excellent with no complaints or device-related adverse events. We found that continuous wrist actigraphy could be used to detect impaired locomotion, which preceded clinical detection of GBM progression with MRI in 2 patients. It will be important to assess the sensitivity and specificity of wrist actigraphy for detecting early progression and recurrence. This could guide changes in treatment for patients with GBM, such as secondary resections, reirradiation, or enrollment in clinical trials. Further, although no effective treatments exist to this date for recurrent GBM, continuous monitoring of progression could contribute further as new therapeutic approaches emerge.

There are multiple limitations to the data collection, analysis, and interpretation of this study. Although results in mice and a small number of patients indicate that daily activity decreases with GBM progression, studies aimed at evaluating this metric as a function of tumor type, location, genotype, and extent of resection in larger and more diverse patient populations (e.g., age, sex, chronotype, and occupation) are needed to validate the sensitivity and specificity of actigraphy-based monitoring of brain cancer. For example, we report data on 6 patients with GBM, 5 of whom were women, which does not capture the known sex differences in GBM incidence and outcomes. Although most patients in this study received either gross total resection or subtotal resection, which correlates with patient survival and disease progression (43), future larger studies must also control for potential confounding variables, including surgery type; age at diagnosis; *MGMT* methylation status, which correlates with response to TMZ; tumor mutational profile (i.e., *ATRX* and *PTEN* expression); additional treatments (i.e., dexamethasone use, tumor-treating fields, prescription of other drugs); individual baseline activity levels; life events; and adverse events related to treatment and disease status.

Another limitation of this study is that patients were required to come into the clinic to download their data. Recent advancements in wearable technology and mobile applications present promising approaches that would allow caregivers access to the data during continuous collection and may reveal noninvasive biomarkers for disease status and treatment efficacy (44–47). This could support more timely informed decisions regarding treatment (48). To complement highly sensitive, interval-scheduled MRI scans, daily activity tracking may provide useful continuous updates on patient status and assist in personalized treatment planning and prognosis determination for each patient. We found actigraphy declined with progression of diverse GBM cell lines implanted in mice of both sexes, with and without chemotherapy, and with and without an intact immune system. Future studies should evaluate whether each patient’s activity and sleep patterns can be used as a guide for personalized interventions (e.g., timing of TMZ administration) to enhance the effectiveness of the standard of care. We further found that patients without disease progression had higher wrist activity counts than those who progressed during study, highlighting its potential use as a prognostic biomarker. Future studies should evaluate whether baseline activity measurements can serve as a biomarker for disease progression and recurrence.

The finding that daily activity declines with tumor growth in preclinical GBM models opens the doors to a deeper mechanistic exploration of how disruption of daily locomotion arises with disease progression. Gliomas can cause local and distal disruption of neuronal functional connectivity as they grow, including critical brain regions responsible for motor control like the motor cortex, suggesting that GBM may disrupt locomotor behaviors as it integrates into neural circuits implicated in motor function (49–52). Moreover, brain tumors have been found to induce neuroinflammation as they grow, and radiation also promotes an inflammatory state (53). One phenotype that arises with neuroinflammation in animal models is a decrease in locomotion, suggesting that an inflammatory state triggered by GBM growth and treatment may explain the observed decrease in daily activity (54, 55). Another potential mechanism by which GBM may disrupt locomotion could be by disrupting and invading white matter tracts, which may hinder efficient transmission of motor signals (56, 57).

In addition, we found that daily wake and rest times, circadian amplitude, and SRI change late in GBM disease, suggesting impairment of the circadian system just before death. Curiously, although mice woke earlier and initiated rest later, our pilot human data showed an opposite trend, with earlier bedtimes and later awakenings. It is possible that these differences come from progression-induced circadian disruption manifesting differently in mice and humans. Previous studies have reported that when daily rhythms are disrupted, greater variability can be seen in sleep-wake patterns in humans, as evidenced by insomnia or excessive sleepiness in patients with GBM (58). It is also possible that circadian disruption manifests differently in mice versus humans given the strict environmental controls where mice are housed in the laboratory setting (i.e., temperature, light intensity, and light duration controls).

Although circadian and sleep disruption is a common symptom in GBM, no studies have addressed how tumor growth disrupts circadian behaviors. Recent studies have found that GBM integrates into the circadian circuit of the brain to grow (32), suggesting that this integration may impair outputs of the circadian system like daily rhythms in locomotion. In addition to preclinical in vitro and in vivo studies showing circadian differences in TMZ efficacy, 2 independent retrospective analyses found time-of-day effects of TMZ on progression-free survival in *MGMT*-unmethylated patients with GBM (59, 60). Notably, they differed in their definition of morning (i.e., 6:00 to 10:00 am vs. midnight to 11 am) and only one found time-of-day effects in *MGMT*-methylated patients and on overall survival. Because TMZ efficacy has been shown to be maximized when delivered exactly at peak expression of the core clock gene *Bmal1* in preclinical models (36), it will be important to measure circadian phase in individual patients to narrow down the best times of day for personalized TMZ treatment. The use of actigraphy in conjunction with a prospective trial of chronotherapy at different times in the patients’ circadian day could improve our understanding of circadian rhythms in individual patients, TMZ efficacy, and GBM progression. Future research should also explore how distinct tumor locations, genotypes, and surgical approaches differentially affect daily rhythms in locomotion. It will be important to learn how GBM growth disrupts motor circuits and circadian behaviors to elucidate potential targets for diagnosis, disease monitoring, and treatment. Because many circadian characteristics change at the time of detection of progression, it will also be important to learn whether these changes are confounded by mental health status upon learning of progression.

Tracking daily activity may be a valuable tool for detecting progression of other diseases beyond GBM. Wrist actigraphy predicts survival of patients with breast, liver, or lung cancer, suggesting that this tool could be used to monitor patients with other cancer types (27–31). Beyond monitoring cancer progression, early locomotor activity disruption may correlate with progression of other diseases, especially neurological disorders characterized by motor impairment (61, 62). Neurodegenerative disorders such as Parkinson’s and Alzheimer’s disease typically manifest in gradual declines in motor and cognitive functions, which could be effectively monitored with daily activity tracking (61, 63, 64). By employing wearable technologies, health care providers could obtain continuous, real-time data that offers insights into the health status of individuals across a spectrum of diseases, enabling proactive management and personalized treatment adjustments.

We propose that as GBM tumors grow, brain regions that control motor behaviors become impaired by the tumor, resulting in a decline in daily locomotion. Tracking this daily behavior is associated with GBM progression in preclinical models and declines before detection of progression by routine MRI scans in patients. Our results establish the feasibility of tracking daily activity in patients and offer an opportunity to assess whether implementing these technologies can provide deeper insights into disease dynamics, facilitate timely personalized interventions that better monitor progression, and improve the treatment and quality of life for patients with GBM.

## Methods

### Sex as a biological variable

This study examined male and female animals, and similar findings are reported for both sexes.

### Glioblastoma cell culture

#### *GL261*.

GL261 cells (obtained from the Division of Cancer Treatment and Diagnosis Tumor Repository of the National Cancer Institute), a male murine model of GBM, were cultured in monolayer in coated T-75 flasks (Nunc EasYFlasks, Thermo Fisher Scientific) using RPMI-1640 media (Sigma-Aldrich), supplemented with 10% FBS, 1% L-glutamine, and 1% penicillin-streptomycin (all Thermo Fisher Scientific). Cells were grown in a 37°C incubator with a 5% CO___2___ environment. Passage numbers in all experiments ranged from 4 to 8. Cells were routinely screened for mycoplasma contamination before xenografting.

#### *Nf1^^^–/–^^^DNp53*.

Nf1^^^–/–^^^DNp53 male astrocytes (gift of Josh Rubin, Department of Neuroscience, Department of Pediatrics, St. Louis Children’s Hospital), a murine model of GBM (64), were cultured in monolayer in coated T-75 flasks (Nunc EasYFlasks, Thermo Fisher Scientific) using 10 mL DMEM/F12 media (Gibco), supplemented with 10% FBS and 1% penicillin-streptomycin (both Thermo Fisher Scientific). Cells were grown in a 37°C incubator with a 5% CO___2___ environment. Passage number in all experiments ranged from 4 to 8. Cells were routinely screened for mycoplasma contamination before xenografting.

#### *B165*.

Human B165 (*MGMT* methylated, male), gift of Albert Kim (Department of Neurosurgery, The Brain Tumor Center, Siteman Cancer Center, Washington University School of Medicine), were cultured as spheres in 100 mm uncoated petri dishes (Thermo Fisher Scientific) using 3 mL DMEM/F12 GlutaMAX media (Gibco), supplemented with 1% penicillin-streptomycin (Thermo Fisher Scientific), 2% B-27 (Miltenyi Biotec), 2.5 mg/mL heparin (Sigma-Aldrich), 20 ng/mL EGF (PeproTech), and 20 ng/mL bFGF (PeproTech). Cells were grown in a 37°C incubator with a 5% CO___2___ environment. Passage number in all experiments ranged from 6 to 10. Cells were routinely screened for mycoplasma contamination before xenografting.

#### *LN229*.

LN229 (American Type Culture Collection), a female human cell line, were cultured in monolayer in coated T-75 flasks (Nunc EasYFlasks, Thermo Fisher Scientific) using 10 mL DMEM/F12 GlutaMAX media (Gibco), supplemented with 5% FBS and 1% penicillin-streptomycin (both Thermo Fisher Scientific). Cells were grown in a 37°C incubator with a 5% CO___2___ environment. Passage number at implant ranged from 4 to 8. Cells were routinely screened for mycoplasma contamination before xenografting.

### Animals

#### *C57BL/6NJ*.

All experiments for orthotopic xenografting of murine GBM cells used 10-week-old immunocompetent male and female *C57BL/6NJ* mice (The Jackson Laboratory, strain 000664). Mice were singly housed in standard 12-hour light/12-hour dark conditions in individual wheel-cages to record locomotor activity in 1-minute bins. After surgery, mice were monitored and treated with analgesic for 4 days. At the end of all experiments, mice were euthanized in accordance with IACUC protocols.

#### *CrTac:Ncr-Foxn1nu*.

All experiments for orthotopic xenografting of human and primary GBM cells used 10-week-old immunocompromised male and female *CrTac:Ncr-Foxn1nu* mice (Taconic, strain NCRNU). Mice were singly housed in standard 12-hour light/12-hour dark conditions in individual wheel-cages to record locomotor activity in 1-minute bins. After surgery, mice were monitored and treated with analgesic for 4 days. At the end of all experiments, mice were euthanized in accordance with IACUC protocols.

### Experimental cell culture

GBM cells were grown to confluence in supplemented media conditions previously mentioned, grown in a 37°C incubator with a 5% CO___2___ environment. Confluent cultures were kept for up to 4 weeks with media refreshed every 3–5 days. Once cultures reached 80% confluence, cells were split using TrypLE Express Enzyme (1×) (Gibco), centrifuged for 4 minutes at 0.4 RCF, diluted, and replated at a lower confluence in new cell culture flasks.

### Orthotopic xenografting

First, 200,000 GBM cells expressing a *Per2*-luciferase reporter (32, 36) were stereotactically implanted into the right caudate putamen (coordinates: Bregma, 2 mm right laterally, 3 mm ventral) of 10-week-old male and female *C57BL/6NJ* (The Jackson Laboratory, strain 005304) mice. Mice were housed in individual cages, monitored, and treated with analgesic for 4 days after implant. Mice were allowed to recover, and in vivo bioluminescence imaging started 4 days after engraftment to measure tumor size. Murine GL261 cells implanted into *C57BL/6NJ* mice model an immune-competent, quickly progressing, aggressive tumor, with a survival of 3–4 weeks (65). Human LN229 cells implanted into *CrTac:Ncr-Foxn1nu* nude mice model an immunocompromised, infiltrative, quickly progressing tumor, with a survival of 6–8 weeks (65).

### Sham surgeries

To control for the effects of orthotopic xenografting on locomotor activity behavior in mice, sham mice (10-week-old male and female, *C57BL/6NJ* or *CrTac:Ncr-Foxn1nu* mice) received 1× PBS stereotaxically injected into the right caudate putamen (coordinates: Bregma, 2 mm right laterally, 3 mm ventral). Mice were housed individually in cages, monitored, and treated with analgesic for 4 days after implant.

### In vivo bioluminescence imaging

Following orthotopic xenografting, tumor size was measured in mice anesthetized with 2% isoflurane and injected subcutaneously with 15 mg/mL of D-luciferin. After 10 minutes, we imaged bioluminescence (Lumina III, IVIS, PerkinElmer, 5-minute exposure). Bioluminescence images were analyzed using Living Image Software (PerkinElmer).

### Body weight measurements

To assess tumor burden and disease progression, mice were weighed every other day starting 7 days before tumor implant. To avoid spontaneous death, which begins to occur around 4 weeks after implantation (66), we euthanized mice at around 25 days after implant in accordance with IACUC protocols.

### Locomotor activity recording and analyses

Mice were singly housed on a 7:00 am-7:00 pm light cycle in wheel-cages to record locomotor activity in 1-minute bins continuously for 1 week before and after tumor implant until endpoint. For each mouse, daily total activity counts, wake onset time, and rest onset time were measured using ClockLab (version 6.1.02, ActiMetrics Software). We measured SRI and circadian amplitude using custom Python code. Wake and rest onset times were determined based on the time of day when mice began or stopped running on a wheel, respectively, for more than 30 minutes. Circadian amplitude was calculated by passing each day’s activity count time series through a limit cycle model of the circadian clock (67), looping until the initial conditions converged, and then extracting the amplitude variable on that day. SRI was approximated by calculating the probability of a mouse being at rest (i.e., counts equal to zero) or active (i.e., counts greater than zero) at the same clock time on consecutive days, with scores ranging from 0 to 100 (68). An SRI closer to 0 indicates irregular sleep patterns (i.e., more variable day to day), and values closer to 100 indicate regular sleep patterns (i.e., less variable day to day).

To correlate each of the 5 metrics of daily rest-activity (i.e., daily average activity, wake onset time, rest onset time, circadian amplitude, and SRI) with body weight and tumor size, we used the Spearman’s rank correlation coefficient test to determine the strength and direction of association between daily measurements. We then performed a nonlinear regression analysis for each pair exhibiting a significant correlation. Two-way repeated-measures ANOVA was applied to model longitudinal metrics (i.e., locomotor activity measurements, body weight) over time for the experimental group effect (i.e., sham vs. GBM, TMZ vs. vehicle) and time effect, while accounting for repeated measures of the same animal. All statistical analyses were conducted in GraphPad Prism (version 10.3.0).

### TMZ gavage in vivo

To analyze the effects of chemotherapy on locomotor activity, we evaluated wheel-running in mice treated with vehicle or TMZ in the morning or evening. Tumor size and body weight data from these mice were described in a prior publication (36). Briefly, we delivered 100 mg/kg TMZ (final volume of 100–200 mL in 1× PBS based on mouse weight with <10% hydroxypropyl methylcellulose) or vehicle by gavage to anesthetized mice (2% isoflurane) at either 11:00 am (Zeitgeber time, ZT 4) or 6:00 pm (ZT11) for 5 consecutive days starting 11–13 days after implant.

### Analysis of wrist actigraphy recordings

Participants were approached for the study during their scheduled clinic visits. Ten patients enrolled in the study received and continuously wore a wrist actigraphy watch. Four patients were excluded from analysis given a non-GBM diagnosis (i.e., 2 patients diagnosed with *IDH* mutant gliomas, 1 with astrocytoma, and 1 with anaplastic oligodendroglioma). Here, we report on the 6 patients diagnosed with primary GBM and who continuously wore an Actiwatch (ActTrust AT0503, Condor Instruments) after completing surgery and during maintenance TMZ treatment. At each monthly visit, patients exchanged their worn Actiwatch for a replacement. Patients wore their Actiwatch for a cumulative average of 22 months (range 8–49 months), corresponding to their length of time in the study. Upon detection of progression, patients returned the watch, ceased TMZ treatment, and left the study to pursue additional treatment options. Of the 6 patients with primary GBM, 2 progressed while wearing an actigraphy watch and 4 remained stable.

Date, time, wrist movements (proportional integral mode), and light exposure (lux) were exported in 5-minute bins. Monthly actigraphy records for each patient were concatenated and annotated with date of progression, as well as start and end of TMZ therapy. For each patient with GBM and through their duration in the study, we analyzed total daily activity counts, wake onset time, rest onset time, circadian amplitude, and SRI from wrist proportional integral mode data. Time series change point detection analysis was performed to identify when locomotor activity measurements significantly declined in individual patient actigraphy records (69).

### Statistics

SRI and circadian amplitude were calculated using previously published custom Python code. Data are presented as mean ± SEM. Pearson and Spearman’s rank correlation coefficient tests were performed to determine the strength and direction of association between daily measurements in mice. In humans, time series change point detection analysis was performed to identify changes in wrist activity in individual patient actigraphy records. Statistical significance of mean differences was determined by either 1-way ANOVA with multiple-comparison test or 2-way ANOVA with multiple-comparison test. The sample size (*n*), statistical analysis, and multiple-comparison test used are reported within the figures or legends for each experiment. A level of *P* less than 0.05 was used to designate significant differences. All statistical analyses were performed using GraphPad Prism (version 10.3.1).

### Study approval

All animal procedures were performed with approval of the Washington University IACUC (animal protocol 23-0105). All procedures performed with human participants were in accordance with the ethical standards of the 1964 Declaration of Helsinki and its amendments and approved by the Washington University in St. Louis IRB. All participants signed a written informed consent form as part of a prospective clinical trial evaluating feasibility and side effects of administering TMZ in the morning or evening at the Washington University Siteman Cancer Center in Saint Louis, Missouri, from June 2016 to August 2020 (ClinicalTrials.gov NCT02781792).

### Data availability

All data, code, and any additional information required to reanalyze the data reported in this paper will be shared by the corresponding author upon request. All Supporting data values associated with the main manuscript and supplemental material are provided in the Supporting Data Values file.

## Author contributions

All authors contributed to the study conception and design. MFGA, SVS, ARD, and OJW conducted animal experiments and analysis. MFGA, ARD, RGNK, GRT, OHB, JLC, JL, JBR, and OJW performed patient data collection and analysis. The first draft of the manuscript was written by MFGA, OJW, and EDH. All authors read and approved the final manuscript.

## Funding support

The clinical trial (NCT02781792) was supported by the Research Fund from the Division of Oncology, Washington University in St. Louis, The Alvin J. Siteman Cancer Center Siteman Investment Program through funding from The Foundation for Barnes-Jewish Hospital and The Barnard Trust, the Children’s Discovery Institute, and Siteman Cancer Center Early Phase Clinical Research Support.

- The authors were supported by the following NIH grants: National Institute of Neurological Disorders and Stroke R21NS120003 and R01NS134885 (EDH and JR). The authors were also supported by the Washington University Siteman Cancer Center.
- This work is the result of NIH funding, in whole or in part, and is subject to the NIH Public Access Policy. Through acceptance of this federal funding, the NIH has been given a right to make the work publicly available in PubMed Central.
- NCI Cancer Center Support Grant P30 CA091842 (PI Eberlein) to Siteman Cancer Center.

## Supplementary Material

Supplemental data

Supporting data values

## Version 1 11/25/2025

In-Press Preview

## Version 2 01/09/2026

Electronic publication
