# Supplementary material for: Daily locomotor activity declines with tumor growth and disease progression in glioblastoma
Source: JCI Insight. 2025 Nov 25;11(1):e194582. doi: 10.1172/jci.insight.194582 (PMC12890484; doi:10.1172/jci.insight.194582)
Supplement: Supplemental data [file jciinsight-11-194582-s134.pdf]

Title: Daily locomotor activity declines with tumor growth and disease progression in glioblastoma

Authors: Maria F. Gonzalez-Aponte[1], Sofia V. Salvatore[1], Anna R. Damato[1], Ruth G.N. Katumba[2], Grayson R. Talcott[2], Omar H. Butt[2], Jian L. Campian[3], Jingqin Luo[4,5], Joshua B. Rubin[6,7], Olivia J. Walch[8,9], Erik D. Herzog[1*]

Affiliations:

[1] Department of Biology, Division of Biology and Biomedical Sciences, Washington University in St. Louis, St. Louis, MO, USA

[2] Division of Oncology, Department of Medicine, Washington University School of Medicine, St Louis, MO, USA

[3] Department of Oncology, Mayo Clinic, Rochester, MN, USA

[4] Department of Surgery, Division of Public Health Sciences, Washington University School of Medicine, St. Louis, MO, USA

[5] Siteman Cancer Center Biostatistics Shared Resource, Division of Public Health Sciences, Department of Surgery, Washington University School of Medicine, St. Louis, MO, USA

[6] Department of Pediatrics, St. Louis Children's Hospital, Washington University School of Medicine, St. Louis, MO, USA

[7] Department of Neuroscience, Washington University School of Medicine, St. Louis, MO, USA

[8] Arcascope Inc., Arlington, VA, USA

[9] Department of Neurology, University of Michigan, Ann Arbor, MI, USA

*Corresponding author:

- Name: Erik D. Herzog
- Address: Washington University in St. Louis, Campus Box 1137, One Brookings Drive, St. Louis, MO 63130-4899
- Telephone number: (314) 935-8635
- E-mail address: herzog@wustl.edu

Supplementary Data

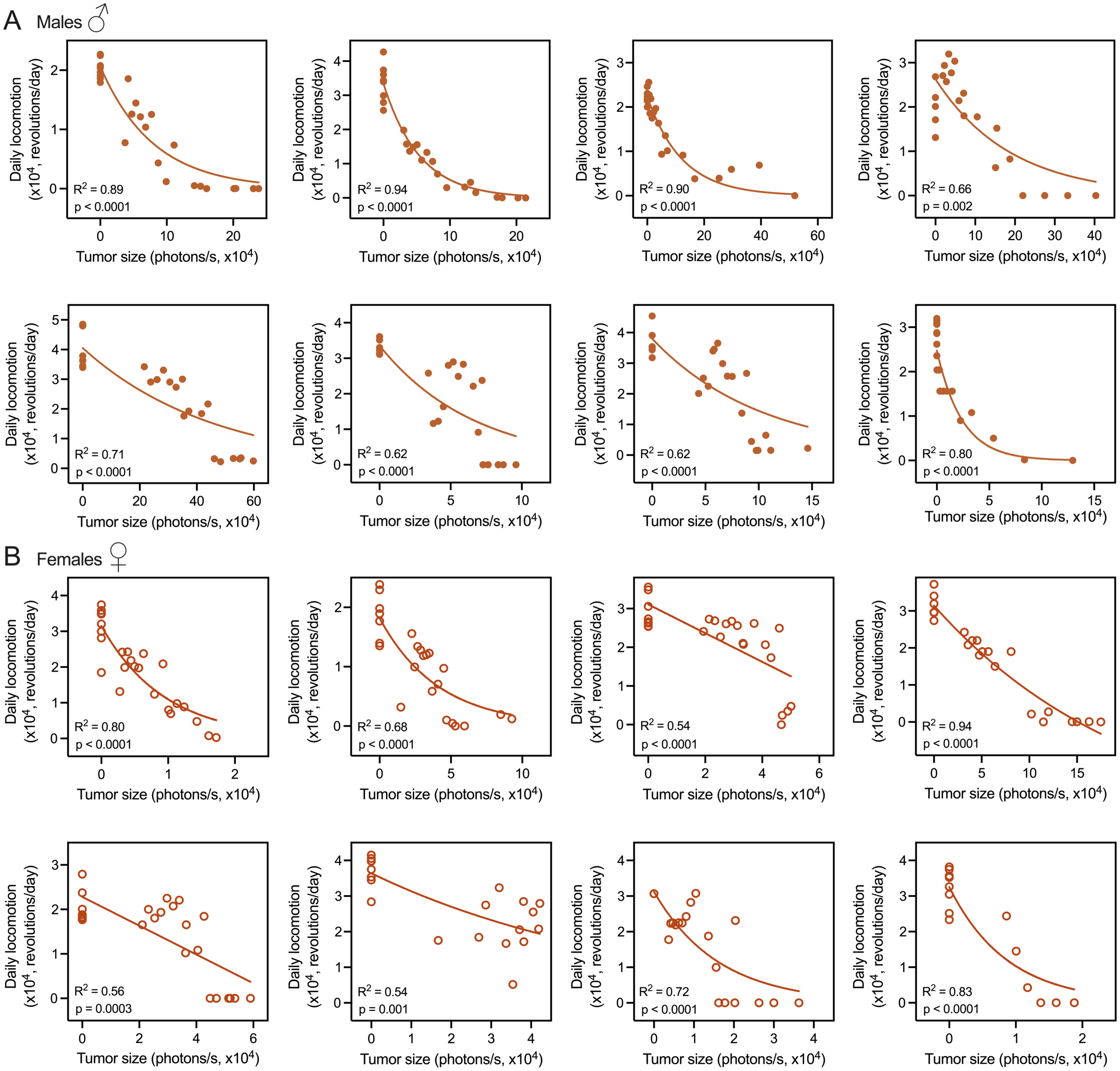

Supplemental Figure 1: Daily locomotor activity decline significantly correlates with tumor growth in GBM-bearing mice, Related to Figure 1.

A) Individual correlations between daily locomotor activity and tumor size in 8 male hosts bearing GBM tumors. All male mice implanted with GBM tumors show significant, non-linear, negative correlations, suggesting that as GBM tumors grew, hosts' running activity declined. Each plot represents one male GBM-bearing mouse, and each dot represents one recording day, excluding the day of surgery and the following recovery days (non-linear regression analysis reported in figure).

B) Individual correlations between daily locomotor activity and tumor size in 8 female hosts bearing GBM tumors. All female mice implanted with GBM tumors show significant, non-linear, negative correlations, suggesting that as GBM tumors grew, hosts' running activity declined. Each plot represents one female GBM-bearing mouse, and each dot represents one recording day, excluding the day of surgery and the following recovery days (non-linear regression analysis reported in figure).

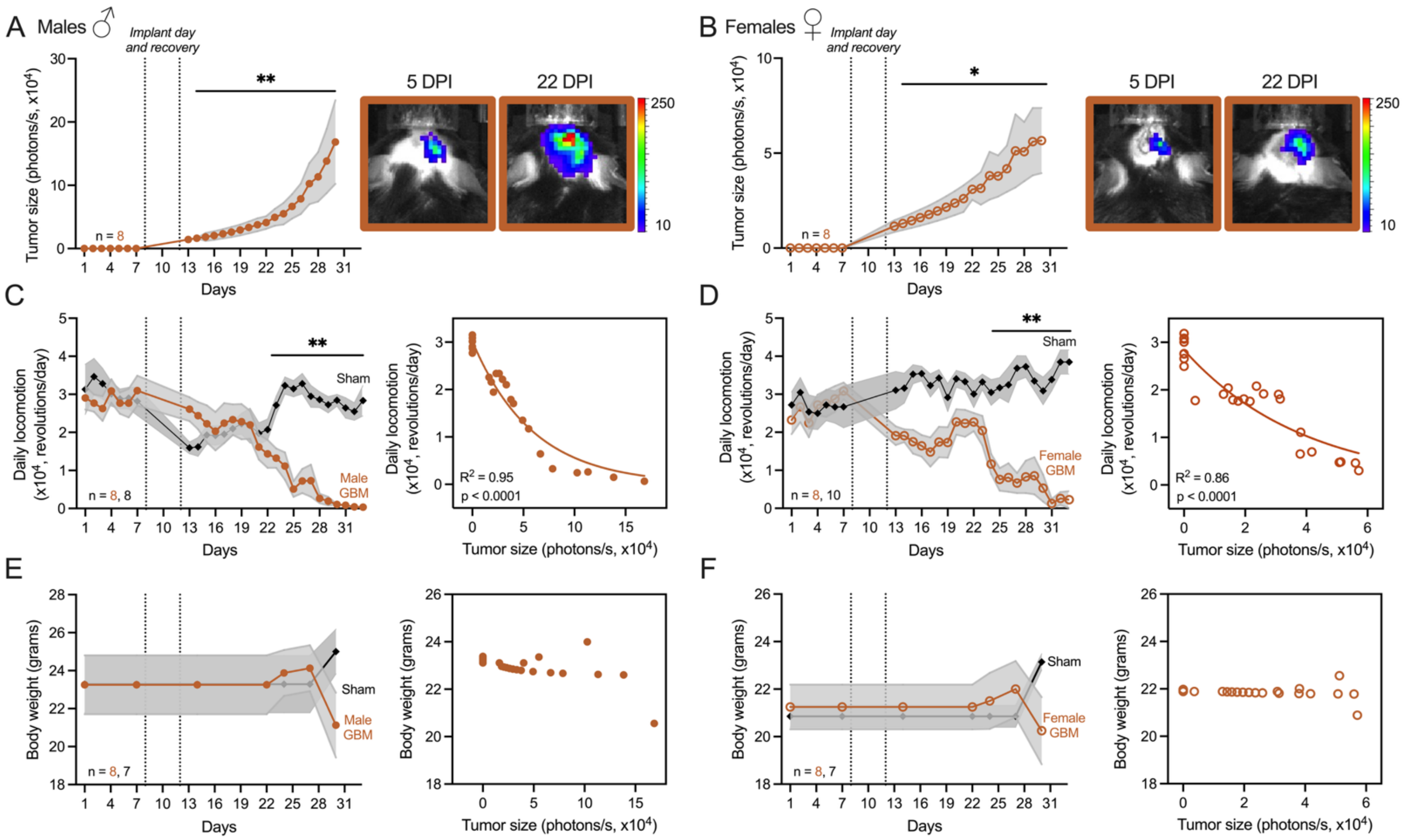

Supplemental Figure 2: Decrease in daily locomotor activity correlates with increase in GBM growth in both males and females *in vivo*, Related to Figure 1.

A) GBM tumor bioluminescence increased by 12-fold from 5 to 22 days post-implant in male mice bearing GL261 tumors (mean±SEM, n reported in figure, one-way repeated measure ANOVA with Tukey's multiple comparisons test, $^{**}p < 0.01$. BLI counts on the representative image are $x10^3$, color bar depicts relative bioluminescence).

B) GBM tumor bioluminescence increased by 5-fold from 5 to 22 days post-implant in female mice bearing GL261 tumors (mean±SEM, n reported in figure, one-way repeated measure ANOVA with Tukey's multiple comparisons test, $^{*}p < 0.05$. BLI counts on the representative image are $x10^3$, color bar depicts relative bioluminescence).

C) *(Left)* Daily locomotor activity of male GBM-bearing mice significantly declined on day 23, corresponding to 15 days post-implant, compared to male sham controls (mean±SEM, n reported in figure, two-way repeated measures ANOVA with Tukey's multiple comparisons test, $^{**}p < 0.01$ from days 23 to 33). *(Right)* Daily locomotor activity negatively correlated with tumor size in male hosts. Each dot represents the average of all male mice per recording day, excluding the day of surgery and the following recovery days (Spearman's correlation, r = -0.9618, 95% confidence interval -0.9837 to -0.9120, $p < 0.0001$, non-linear regression analysis reported in figure).

D) *(Left)* Daily locomotor activity significantly declines in female GBM-bearing mice on day 24, corresponding to 16 days post-implant, compared to female sham controls (mean±SEM, n reported in figure, two-way repeated measures ANOVA with Tukey's multiple comparisons test, $^{**}p < 0.01$ from days 24 to 33). *(Right)* Daily locomotor activity negatively correlated with tumor size in female hosts. Each dot represents the average of all female mice per recording day, excluding the day of surgery and the following recovery days (Spearman's correlation, r = -0.8953, 95% confidence interval -0.9535 to -0.7729, $p < 0.0001$, non-linear regression analysis reported in figure).

E) *(Left)* Body weight does not significantly change in male tumor-bearing mice, compared to sham controls (mean±SEM, n reported in figure, two-way ANOVA with Tukey's multiple comparisons test, ns $p > 0.05$).

(Right) Body weight does not correlate with tumor size in male mice bearing GBM tumors (Spearman's correlation, r = -0.4636, 95% confidence interval -0.3820 to -0.2322, p = 0.0651).

F) *(Left)* Body weight does not significantly change in female tumor-bearing mice, compared to sham controls (mean±SEM, n reported in figure, two-way ANOVA with Tukey's multiple comparisons test, ns p > 0.05). *(Right)* Body weight does not correlate with tumor size in females bearing GBM tumors (Spearman's correlation, r = -0.2723, 95% confidence interval -0.5967 to 0.1286, p = 0.1784).

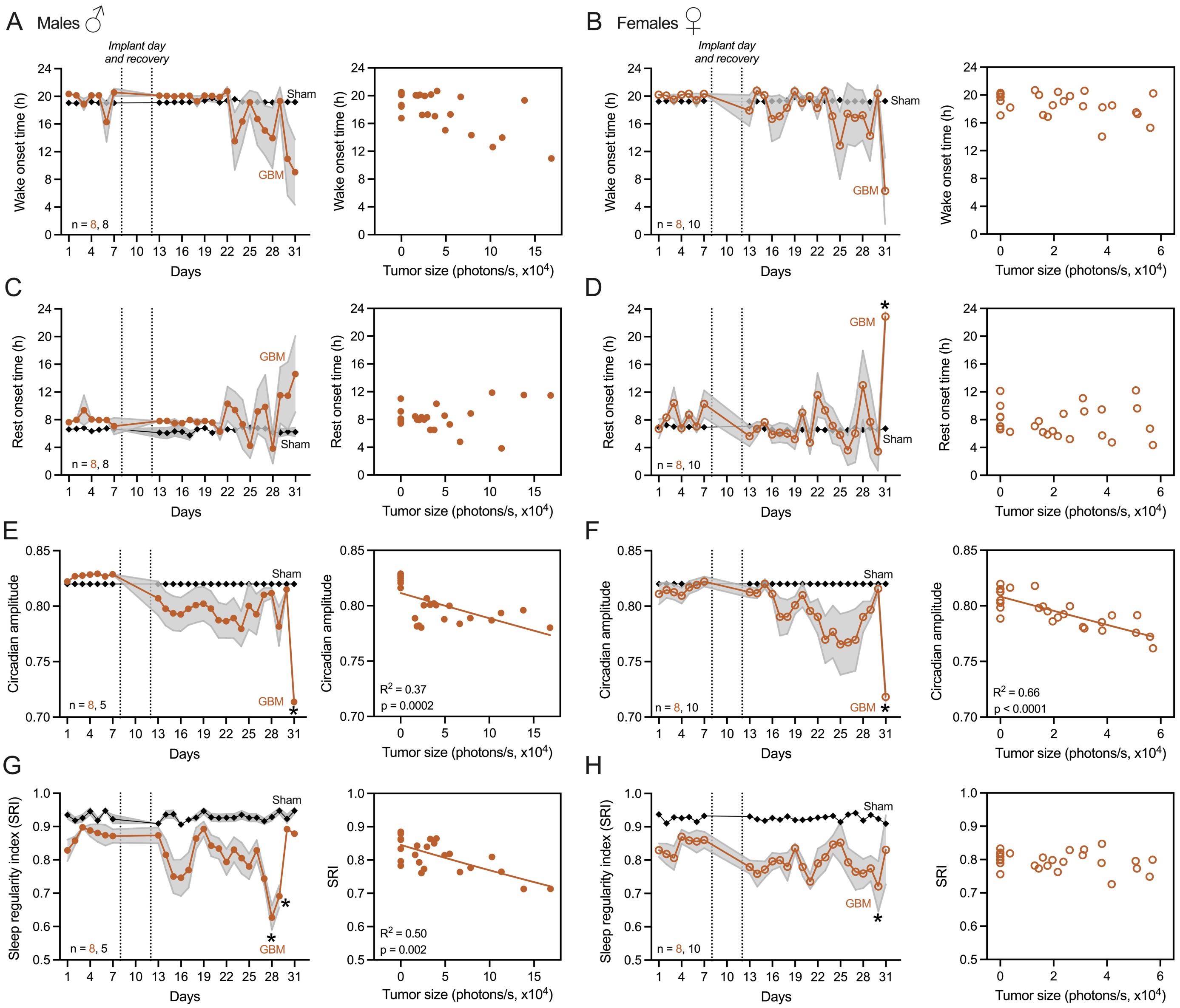

Supplemental Figure 3: Daily wake-up and rest time, circadian amplitude, and sleep regularity index change late in GBM progression regardless of host sex and tumor growth, Related to Figure 1.

A) *(Left)* Wake onset time becomes more variable and earlier towards the end of disease (day 30 of recording, 22 days post-implant) in male GBM-bearing mice, compared to sham controls (mean±SEM, n reported in figure, two-way ANOVA with Tukey's multiple comparisons test, ns $p > 0.05$). *(Right)* Wake onset time does not correlate with tumor size in males bearing GBM tumors (Spearman's correlation, r = -0.4611, 95% confidence interval -0.5874 to -0.2014, p = 0.3050).

B) *(Left)* Wake onset time becomes more variable and earlier towards the end of disease (day 30 of recording, 22 days post-implant) in female GBM-bearing mice, compared to sham controls (mean±SEM, n reported in figure, two-way ANOVA with Tukey's multiple comparisons test, ns $p > 0.05$). *(Right)* Wake onset time does not correlate with tumor size in females bearing GBM tumors (Spearman's correlation, r = -0.3174, 95% confidence interval -0.6349 to 0.09174, p = 0.1141).

C) *(Left)* Rest onset time becomes more variable and later towards the end of disease (day 30 of recording, 22 days post-implant) in male GBM-bearing mice, compared to sham controls (mean±SEM, n reported in figure, two-way ANOVA with Tukey's multiple comparisons test, ns $p > 0.05$). *(Right)* Rest onset time does not correlate with tumor size in males bearing GBM tumors (Spearman's correlation, r = 0.1325, 95% confidence interval -0.2885 to 0.5106, p = 0.5277).

D) *(Left)* Rest onset time becomes more variable and later towards the end of disease (day 30 of recording, 22 days post-implant) in female GBM-bearing mice, compared to sham controls (mean±SEM, n reported in figure, two-way ANOVA with Tukey's multiple comparisons test, ns $p > 0.05$). *(Right)* Rest onset time does not correlate with tumor size in females bearing GBM tumors (Spearman's correlation, r = -0.1717, 95% confidence interval -0.5329 to 0.2424, p = 0.4016).

E) *(Left)* Circadian amplitude significantly decreases on day 30 of recording (i.e., 22 days post-implant) in male GBM-bearing mice, compared to sham controls (mean±SEM, n reported in figure, two-way ANOVA with Tukey's multiple comparisons test, *$p < 0.05$). *(Right)* Circadian amplitude weakly correlates with tumor size in males bearing GBM tumors (Spearman's correlation, r = -0.6733, 95% confidence interval -0.8474 to -0.3683, p = 0.0002, non-linear regression analysis reported in figure).

F) *(Left)* Circadian amplitude significantly decreases on day 30 of recording (i.e., 22 days post-implant) in female GBM-bearing mice, compared to sham controls (mean±SEM, n reported in figure, two-way ANOVA with Tukey's multiple comparisons test, *$p < 0.05$). *(Right)* Circadian amplitude correlates with tumor size in females bearing GBM tumors (Spearman's correlation, r = -0.7920, 95% confidence interval -0.9047 to -0.5756, $p < 0.0001$, non-linear regression analysis reported in figure).

G) *(Left)* Sleep regularity index (SRI) significantly decreases on day 28 of recording (i.e., 20 days post-implant) in male GBM-bearing mice, compared to sham controls (mean±SEM, n reported in figure, two-way ANOVA with Tukey's multiple comparisons test, *$p < 0.05$). *(Right)* SRI weakly correlates with tumor size in males bearing GBM tumors (Spearman's correlation, r = -0.5837, 95% confidence interval -0.7999 to -0.2335, p = 0.0022).

H) *(Left)* Sleep regularity index (SRI) significantly decreases on day 30 of recording (i.e., 22 days post-implant) in female GBM-bearing mice, compared to sham controls (mean±SEM, n reported in figure, two-way ANOVA with Tukey's multiple comparisons test, *$p < 0.05$). *(Right)* SRI does not correlate with tumor size in females bearing GBM tumors (Spearman's correlation, r = -0.2151, 95% confidence interval -0.5644 to 0.1995, p = 0.2913).

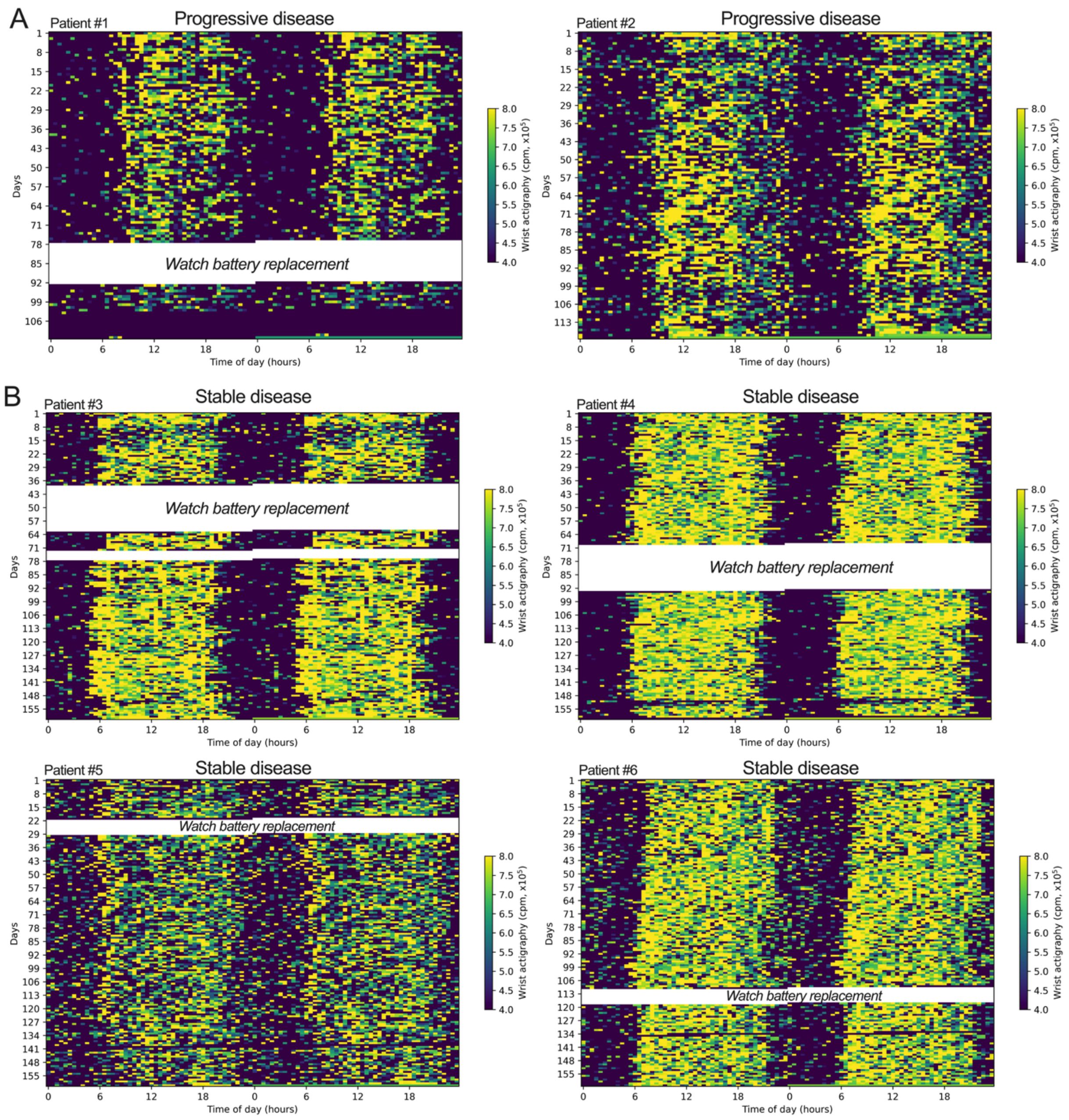

Supplemental Figure 4: Daily wrist activity declined with GBM progression in human patients, Related to Figure 3.

A) Actograms of two GBM patients (Patient #1 and #2) with progressive disease (i.e., tumor size increased on imaging scans during the study) wearing an actigraphy watch after completing surgery and during maintenance TMZ treatment shows a decline in daily activity. Color bar depicts wrist activity counts. White space represents days when actiwatches required a battery replacement and no data was collected.

B) Actograms of four GBM patients (Patients #3-6) with stable disease (i.e., did not progress during study) wearing an actigraphy watch after completing surgery and during maintenance TMZ treatment shows no decline in daily activity. Color bar depicts wrist activity counts. White space represents days when actiwatches required a battery replacement and no data was collected.

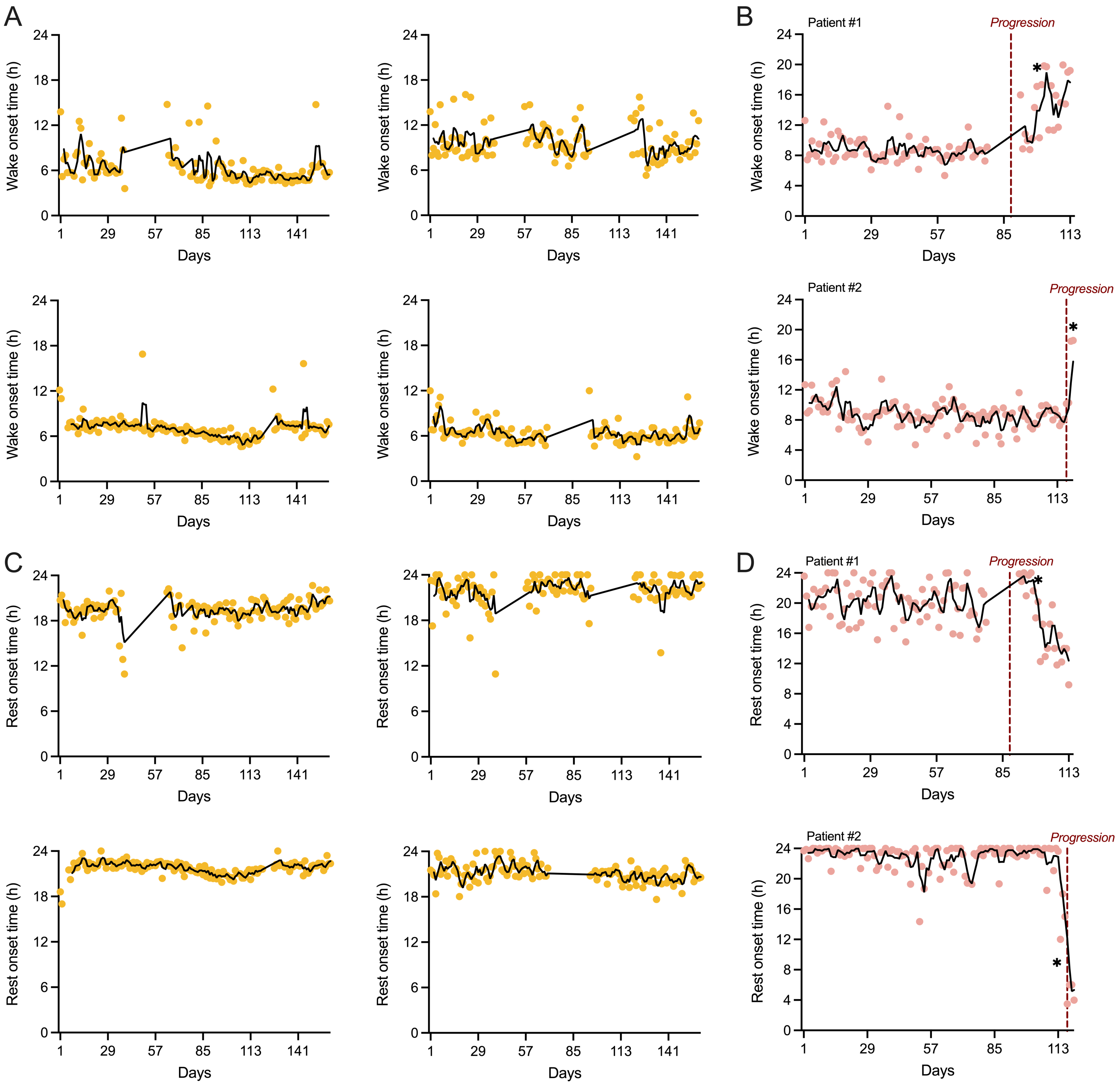
A
B
C
D
Patient #1
Patient #2
Progression
*
Wake onset time (h)
Rest onset time (h)
Days
0
4
6
8
12
16
18
20
24
1
29
57
85
113
141

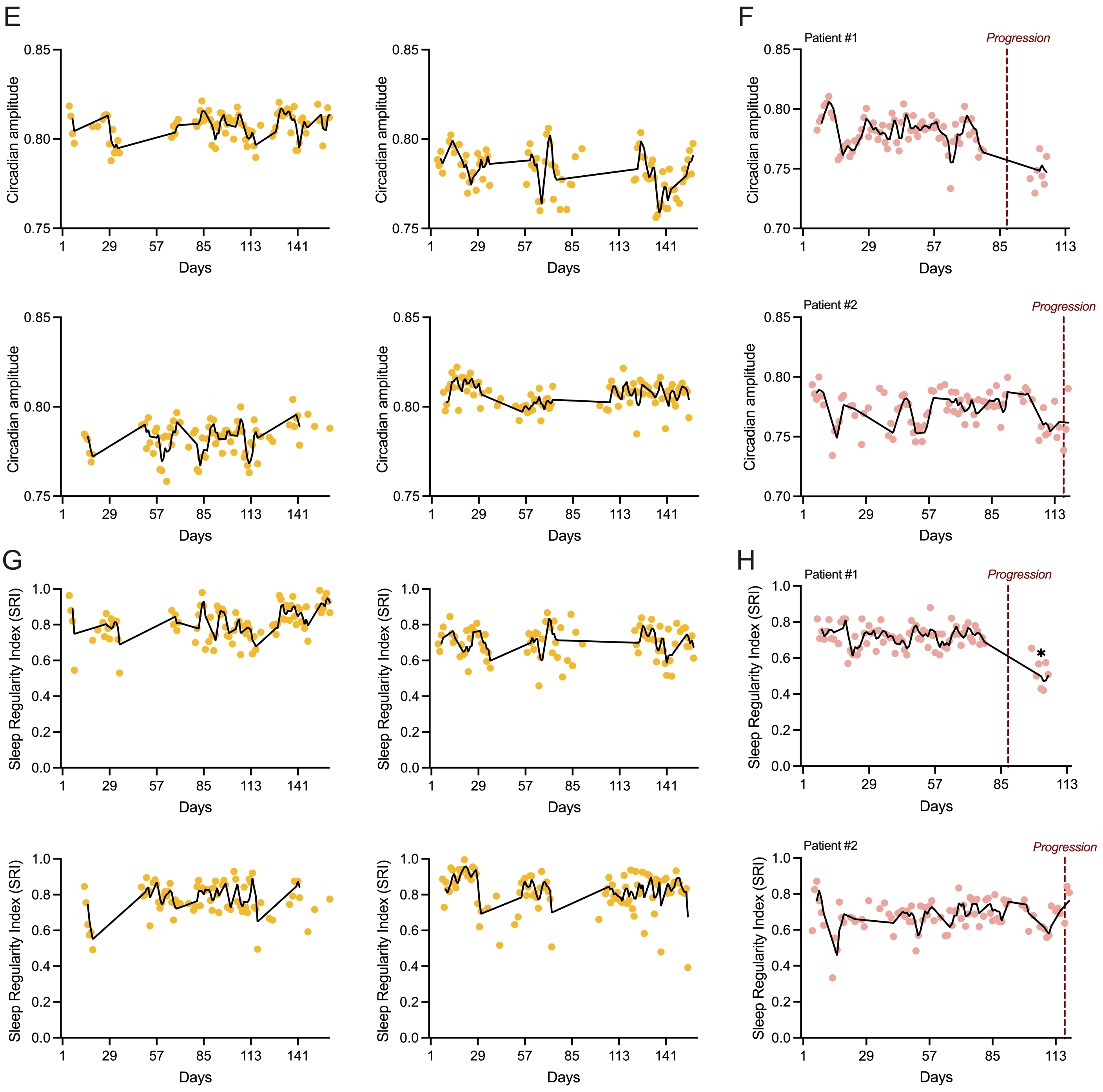

Supplemental Figure 5: Daily wake-up and rest time, circadian amplitude, and sleep regularity index change late with GBM progression in human patients, Related to Figure 3.

A) Wake onset time did not change in four GBM patients with stable disease progression (i.e., did not progress during study, each plot on left and middle columns refers to an individual GBM patient, black line indicates a 3-day running average of daily wake onset data, time series change point detection analysis, ns $p > 0.05$).

B) Daily wake onset time was delayed after detection of progression in two GBM patients who progressed during study (i.e., tumor size increased on imaging scans). Top and bottom plots refer to each GBM patient who progressed (black line indicates a 3-day running average of daily wake onset data, red dashed line indicates date of clinical progression, time series change point detection analysis, *$p < 0.05$).

C) Rest onset time did not change in four GBM patients with stable disease progression (each plot on left and middle columns refers to an individual GBM patient, black line indicates a 3-day running average of

daily rest onset data, time series change point detection analysis, ns $p > 0.05$).

D) Daily rest onset time advanced after detection of progression in two GBM patients who progressed during study. Top and bottom plots refer to each GBM patient who progressed (black line indicates a 3-day running average of daily wake onset data, red dashed line indicates date of clinical progression, time series change point detection analysis, *$p < 0.05$).

E) Circadian amplitude did not change over the course of disease in four GBM patients with stable disease progression (each plot on left and middle columns refers to an individual GBM patient, black line indicates a 3-day running average of daily circadian amplitude, time series change point detection analysis, ns $p > 0.05$).

F) Circadian amplitude did not change over the course of disease in two GBM patients who progressed during the study. Top and bottom plots refer to each GBM patient who progressed (black line indicates a 3-day running average of daily circadian amplitude, red dashed line indicates date of clinical progression, time series change point detection analysis, ns $p > 0.05$).

G) Sleep regularity index (SRI) remained unchanged in four GBM patients with stable disease progression (each plot on left and middle columns refers to an individual GBM patient, black line indicates a 3-day running average of daily SRI data, time series change point detection analysis, ns $p > 0.05$).

H) Sleep regularity index (SRI) significantly decreased in one GBM patient who progressed during the study, but not in a second patient. A significant decline in weekly SRI, defined to be at least a 10% change that did not subsequently increase, was detected on day 103 (Patient #1, 15 days after clinical detection of progression), but was not detected in Patient #2 (black line indicates a 3-day running average of daily SRI data, red dashed line indicates date of clinical progression, time series change point detection analysis, *$p < 0.05$).
